# Scientific Publication Performance of the Erector Spinae Plane Block in Türkiye: A Bibliometric Analysis

**DOI:** 10.4274/TJAR.2023.231432

**Published:** 2023-12-27

**Authors:** Sibel Çatalca, Özlem Özmete, Nesrin Bozdoğan Özyılkan

**Affiliations:** 1 Clinic of Anaesthesiology and Reanimation, Başkent University, Adana Dr. Turgut Noyan Application and Research Center, Adana, Turkey

**Keywords:** Algology, bibliometrics, journal impact factor, pain, postoperative, regional anaesthesia

## Abstract

**Objective:**

Erector spina plane block (ESPB) was first described in 2016 and is effective in various surgical procedures. Bibliometric analysis is a novel method that evaluates the contribution of scientific studies conducted in a specific field on the existing literature. This study examined articles on ESPB published by anaesthesia clinics in Türkiye in journals under the Science Citation Index Expanded (SCI-E) category.

**Methods:**

Studies on ESPB indexed in the Web of Science Core Collection and published in Türkiye from 2018 to 2022 were evaluated. The primary outcome was to determine the number of studies published in journals under the SCI-E category. The secondary aims were to determine the number of citations and the institutions where the studies were conducted.

**Results:**

A total of 159 publications were analyzed. The journal with the highest number of publications was “Journal of Clinical Anesthesia” (n = 70). The institution that has to date made the most contributions to the literature was Atatürk University (n = 31). The most cited article was “Ultrasound guided erector spinae plane block reduces postoperative opioid consumption following breast surgery: A randomized controlled study.” published by Gürkan et al. (n = 175).

**Conclusion:**

This study reflects the contribution level of Türkiye-addressed anaesthesia clinics to journals under the SCI-E category. Our findings can serve as a benchmark for attracting the attention of national and international researchers.

**Main Points**

• The erector spinae plane block (ESPB) has been a popular block in recent years.

• Bibliometric analysis is used to evaluate the contribution of published studies conducted in a specific field on the literature.

• The journal with the highest number of Türkiye-addressed publications on ESPB is the “Journal of Clinical Anaesthesia”.

• The most cited Türkiye-addressed article on ESPB is “Ultrasound guided erector spinae plane block reduces postoperative opioid consumption following breast surgery: A randomized controlled study.” published by Gürkan et al. (2018) in the Journal of Clinical Anesthesia.

## Introduction

Erector spinae plane block (ESPB) provides analgesia in a large dermatomal area by injecting a local anaesthetic agent into the space between the vertebral transverse process and the erector spinae muscle. It has become exceedingly popular in recent years because of its effectiveness, easy application, and low complication rate. ESPB was first performed by Foreo in 2016 in two patients with thoracic neuropathic pain and rib fractures.^1^ Anatomical and radiological studies performed on fresh cadavers have demonstrated that ESPB affects the dorsal and ventral nerve branches in the spinal medulla.^2^ Due to its spread of approximately 3-4 segments cranially and caudally from the site of administration, ESPB has been increasingly used in postoperative pain management and the treatment of neuropathic pain in various procedures. ESPB can be performed at the lumbar, thoracic, cervical, and sacral vertebrate levels.^2,3,4,5,6^

Bibliometric analysis (BA) is a novel method that examines the contribution of scientific studies published in a specific field to the literature through statistical and visual analysis.^7^ In BAs involving medical areas, databases such as Web of Science (WoS), Scopus, Cochrane Library, PubMed, and Google Scholar are frequently used for the evaluation and measurement of scientific outputs.^8,9,10^ One of the important criteria used in this analysis is the citation count. As an article’s citation increase, its impact on the respective field also grows.^11,12^

In recent years, BAs related to anaesthesia have been conducted to assess the contributions and citation counts of publications, authors, institutions, journals, and countries.^8,13^ Several BAs have also previously been performed on regional anaesthesia.^9,14^ However, there is an extremely limited number of BAs that have focused on ESPB.^15^ No analysis evaluating scientific studies from Türkiye on ESPB and published in high-impact factor journals listed in international indexes has been found. Assessing the current situation in Türkiye is necessary for developing research and training institutions. Determining the most cited articles and high-impact factor journals may help researchers review Türkiye-addressed literature and identify new directions while planning future studies. This study evaluated articles on ESPB published by anaesthesia clinics in Türkiye in journals categorized under the Science Citation Index Expanded (SCI-E).

## Methods

The study protocol was approved by the Başkent University Institutional Review Board (approval no: KA23/150). In this study, we collected articles published by anaesthesiologists in Türkiye that focused on ESPB up to 2022 as a data source. The “advanced search” feature of the WoS database was used to identify relevant publications (https://www.webofscience.com/wos/woscc/advanced-search, access date 25.04.2023). A comprehensive search was performed using the terms “erector spinae plane block” or “erector spina plane block” to determine publications from Türkiye. Documents published in journals in the SCI-E category up to December 31, 2022 were filtered. Journals outside the SCI-E category and publications from 2023 were excluded from the analysis. The full search query was as follows: [ALL=(erector spina plane block) or ALL=(erector spinae plane block)) and ADDRESS=(Turkey) I Time span: 2016-01-01 to 2022-12-31 (Publication Date)]. After reading the abstracts of the retrieved publications, those that were not related to ESPB and excluded an anaesthetist in the author list were excluded.

All data were exported to a Microsoft Excel (2003 version) table, including the publication title, publication year, type of publication registered in WoS (article, editorial, letter to the editor, review, conference abstract), the journal in which the article was published, authors’ institutions, cited references in Türkiye-addressed publications, and keywords and citation numbers in WoS. Based on the abstracts, publications that were case reports were categorized as “case report/series”, while publications that involved critiques of articles were categorized as “letter to the editor”. This reclassification was performed according to the content of the publication type, such as case report/series, letter to the editor, review, cadaver study, retrospective study, and randomized controlled study (RCT). Considering the age of the published article, the annual average number of citations was calculated. The 2021 Journal Impact Factors (JIF) were obtained from the Thomson Reuters InCites database (access date 30.4.2023, https://jcr.clarivate.com/jcr/browse-journals). The institution of the first-listed anaesthetist was accepted as the “institution of the first author”. Each article was examined individually to determine the type of pain affected by ESPB (postoperative pain, neuropathic pain), the surgical procedure, the level of the block (cervical, thoracic, lumbar, or sacral), and the vertebral level at which ESPB was applied.

The primary outcome was to determine the number of studies published in journals under the  SCI-E category. The secondary aims were to define publication year, publication type, institutions, keywords, number of citations, type of pain affected by ESPB, surgical procedure, level of block that had been performed, and most frequently cited references in the publications.

The Excel program was used for the mathematical and visual analyses. Visual analysis of the top 19 most cited references in Türkiye-addressed publications, as well as keywords that were used at least five times, was performed using the VOSviewer program (version 1.6.19).

## Results

**Figure 1:**
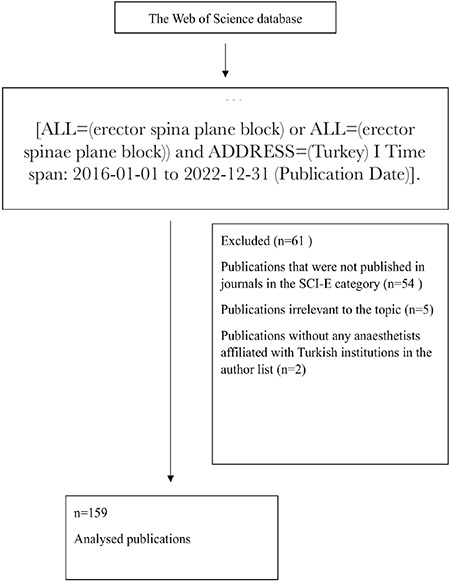
Consort diagram

A total of 220 results related to ESPB in Türkiye were obtained from the WoS database. Among these, 54 publications had not been published in journals in the SCI-E category, 5 were irrelevant to the topic, and 2 were excluded due to no anaesthetists affiliated with Turkish institutions in the author list. Finally, 159 publications met all the criteria for inclusion in the study (Figure 1).

The 159 publications included in the analysis were published in 46 different journals within the SCI-E category. The list of journals in which the studies were published, JIFs, total number of publications in the journals, number of WoS citations, and average citations per publication are presented in Table 1.

It was observed that among the publications in the WoS, 56.6% (n = 90) were in the form of letters to the editor, 37.7% (n = 60) were articles, 3.1% (n = 5) were reviews, 1.2% (n = 2) were editorials, and 1.2% (n = 2) were in the conference abstract category. When the abstracts of the articles were analyzed to determine the type of publication, 47.7% (n = 76) were case reports/series, 27.6% (n = 44) were RCTs, 14.4% (n = 23) were letters to the editor providing criticism/contribution/response to previous studies, 4.4% (n = 7) were retrospective studies, 3.1% (n = 5) were reviews, 1.2% (n = 2) were conference abstracts, 0.6% (n = 1) was cadaver study, and 0.6% (n = 1) was an editorial.

ESPB was applied to patients for pain management in 128 of the 159 publications. It was performed in 111 publications on adult patients, 16 publications on paediatric patients, and 1 publication on both patient groups. When analyzing the vertebral levels at which ESPB was performed, it was found to have been applied at the thoracic level in 98 publications, the lumbar level in 28 publications, and the sacral level in 5 publications. ESPB was used for acute pain management in 94.53% (n = 121) and chronic pain management in 5.78% (n = 7) of these publications.

When analyzing publications related to acute pain management, ESPB was found to have been performed for intraoperative/postoperative pain management in 94.21% of the publications (n = 114), for acute pain management in patients with active Zona Zoster in 3.3% (n = 4), for pain management in the emergency department (renal colic) in 1.65% (n = 2), and for pain management in the intensive care unit (rib fracture) in 0.82% (n = 1). The interventions and vertebral levels at which ESPB was performed for intraoperative/postoperative analgesia are shown in Table 2.

When analyzing publications related to chronic pain management, ESPB was observed to have been performed for the following procedures: myofascial pain syndrome (n = 3), neuropathic pain related to thoracicgynecological and urological malignancies (n = 3), post-herniorrhaphy neuralgia (n = 1), and chronic lumbar disk pain (n = 1).

When examining the annual distribution of the publications, this study found that 10% (n = 16) of the publications were published in 2018, 31.1% (n = 50) in 2019, 22.6% (n = 36) in 2020, 13.2% (n = 21) in 2021, and 22.6% (n = 36) in 2022.

The institution that has made the most contributions to the literature on ESPB in Türkiye to date is Atatürk University (n = 31). This was followed by Maltepe University (n = 27), Kocaeli University (n = 19), Koç University (n = 10), and Medipol University (n = 10). When we reanalyzed the institutions linked to these publications according to the affiliation of the first author, Atatürk University was found to have the highest number of publications in the SCI-E category related to ESPB (n = 18) with a first author (Table 3).

When the citation numbers of the publications were evaluated, it was observed that 159 publications received 2065 citations according to the WoS database. The most cited article was “Ultrasound-guided erector spinae plane block reduces postoperative opioid consumption following breast surgery: A randomized controlled study.” published by Gürkan et al.^4^ in the Journal of Clinical Anesthesia (n = 175). The top 10 most cited publications, the number of citations in the WoS database, and the annual average number of citations are shown in Table 4.^4,16,17,18,19,20,21,22,23^

**Figure 2:**
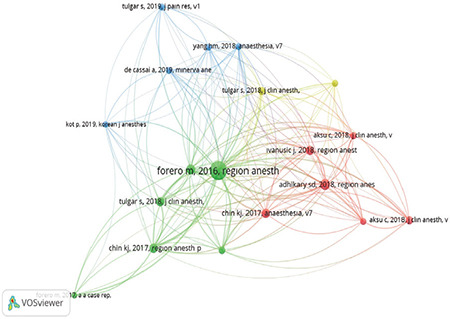
The visual analysis of the top 19 most frequently cited references in Türkiye-addressed publications was created using the VOSviewer program. (Footnote: Each circle is demonstrated by the first author, the year of publication, and the journal in which the cited article was published. The size of the circle is indicated by the number of citations. Colours indicate clustering in the field of erector spinae plane block. The thickness of the lines is related to the co-citations.)

A total of 1283 sources were used in 159 publications. Among these publications, the number of references cited at least 10 times was 19, and the number of references cited at least 20 times was 7. Figure 2 presents a visual analysis of the top 19 most frequently cited references in Türkiye-addressed publications (created using the VOSviewer program). The most cited publications are Forero et al.^1^ (n = 89), Adhikary et al.^24^ (n = 29), Gürkan et al.^4^ (n = 23), Chin et al.^25^ (n = 22), Chin et al.^26^ (n = 21), Tulgar et al.^16^ (n = 20), and Ivanusic et al.^27^ (n = 20).

When the keywords were analyzed, 189 different keywords were identified. It was found that 12 keywords were used 5 or more times and “erector spinae plane block” was the most frequently used keyword (n = 52). Other keywords that were used 5 or more times were postoperative analgesia (n = 26), postoperative pain (n = 15), ultrasound (n = 14), analgesia (n = 13), nerve block (n = 8), pain (n = 8), regional anaesthesia (n = 7), pediatric anaesthesia (n = 6), ultrasonography (n = 6), laparoscopic cholecystectomy (n = 5), and pain management (n = 5).

## Discussion

In this study, Türkiye-addressed publications were identified using the keywords “erector spina plane block” or “erector spinae plane block”. According to the results of our research, the journal with the highest number of Türkiye-addressed publications was the Journal of Clinical Anesthesia. The institution that has made the largest contribution to the literature to date was Atatürk University (n = 31). The most cited publication was an article titled “Ultrasound-guided erector spinae plane block reduces postoperative opioid consumption following breast surgery: A randomized controlled study.” by Gürkan et al.^4^ (n = 175).

In recent years, BAs have become a frequently used method to determine the number and quality of published studies.^7,8^ Chen et al.^28^ which researched the global distribution of studies on anaesthesiology, it was reported that Türkiye ranks seventh worldwide in terms of the number of RCTs published in journals in the SCI-E category (n = 671, 4.78%). Another study emphasized that a country’s level of economic development was set as an important factor in the number of available publications. However, countries such as Türkiye, China, and India have made significant contributions to the literature on anaesthesiology.^29^ Similar to the topic of anaesthesiology Türkiye has made a significant contribution to the literature on regional anaesthesia. In a recent study, Kayir and Kisa^9^ analyzed publications on regional anaesthesia between 1980 and 2019 using the WoS database. The authors reported that the countries with the highest number of articles on regional anaesthesia were the United States (n = 1,583), Germany (n = 585), England (n = 510) and Türkiye (n = 386).

After conducting a literature review, the present study found only one BA related to ESPB. In this analysis, Huang et al.^15^ Evaluated articles published in journals in the SCI-E category between 2016 and July 2022. Similar to our research, this study used WoS as a database. A total of 762 articles were found in this analysis, and Türkiye ranked third (n = 56) after the United States and China, with Atatürk University ranking fourth (n = 10) worldwide in terms of the number of articles. In this BA, similar to our study’s results, the most frequently used keywords were erector spinae plane block, postoperative analgesia, pain management, and postoperative pain. These findings suggest that Turkish anaesthetists used shared keywords and terminology similar to those used in the global literature.

According to the results of our study, the journals with the highest number of publications on ESPB from Türkiye included the Journal of Clinical Anesthesia, Regional Anesthesia and Pain Medicine, and Minerva Anestesiologica. The journals with the highest number of citations per article were the Journal of Pain Research, Journal of Cardiothoracic and Vascular Anesthesia, World Neurosurgery, Anaesthesia Critical Care & Pain Medicine, and Anaesthesist. It is recommended that authors who wish to publish their studies on ESPB in journals with high JIF and to receive more citations should consider these journals as their first choice.

When publications were analyzed based on the total number of citations, the article with the highest number of citations was identified as an RCT published in the “Journal of Clinical Anesthesia” (n = 175).^4^ Furthermore, this article had the highest annual average number of citations (30.6%). When examining the references cited in the publications, Forero et al.^1^ (n = 89), Adhikary et al.^24^ (n = 29), Gürkan et al.^4^ (n = 23), Chin et al.^25^ (n = 22), Chin et al.^26^ (n = 21), Tulgar et al.^16^ (n = 20), and Ivanusic et al.^27^ (n = 20) were identified as the publications with the highest number of citation. Therefore, we suggest that anaesthetists interested in ESPB research should first review these studies.

In this study, the first publication on ESPB from Türkiye was published in 2018.^5^ While the number of publications showed an increasing trend in the first 2 years of the observed period, a significant decrease was detected in 2020. A similar publication curve plot was also available in BA on ESPB published by Huang et al.^15^. We believe that this decrease was likely due to the outbreak of the global coronavirus pandemic.

### Study Limitations

Our study has some limitations. First, the literature search was limited to WoS, and other databases such as Cochrane, PubMed, Google Scholar, and Scopus were not evaluated. Second, only publications in journals in the SCI-E category were included in our study. Publications in journals outside the SCI-E category and in groups such as books and book chapters were not analyzed. Finally, we excluded publications authored by non-anaesthetists.

## Conclusion

This study provides a detailed evaluation of the most influential studies conducted in Türkiye on ESPB. Our findings can help researchers interested in this type of block better understand the situation and identify new directions for future research.

## Figures and Tables

**Table 1:**
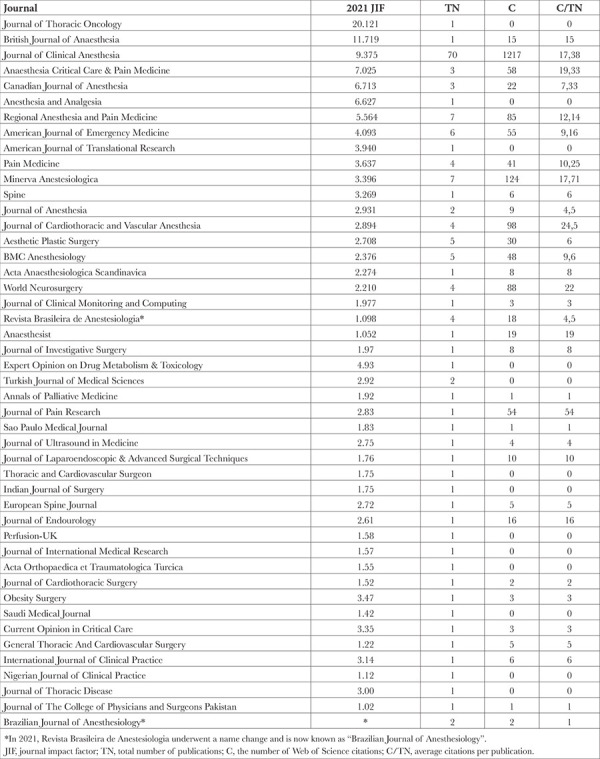
Journals in the Science Citation Index Expanded Category with Publications from Türkiye, Their 2021 Impact Factors, Total Number of Publications in the Journals, the Number of Web of Science Citations, and Average Citations Per Publication

**Table 2:**
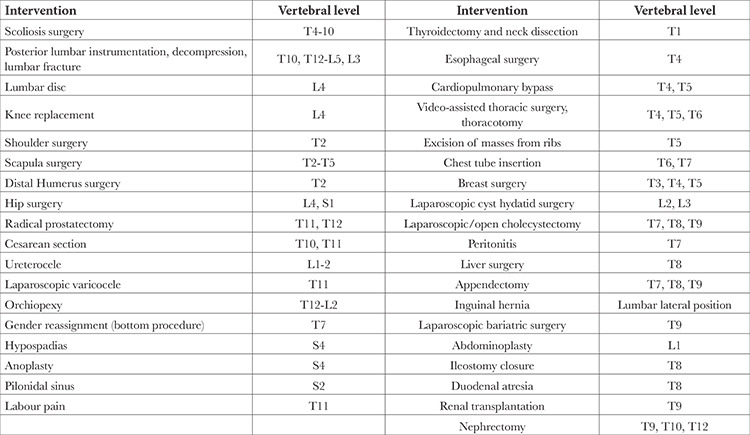
The Interventions and the Vertebra Levels in Which Erector Spinae Plane Block was Performed for Intraoperative/Postoperative Analgesia

**Table 3:**
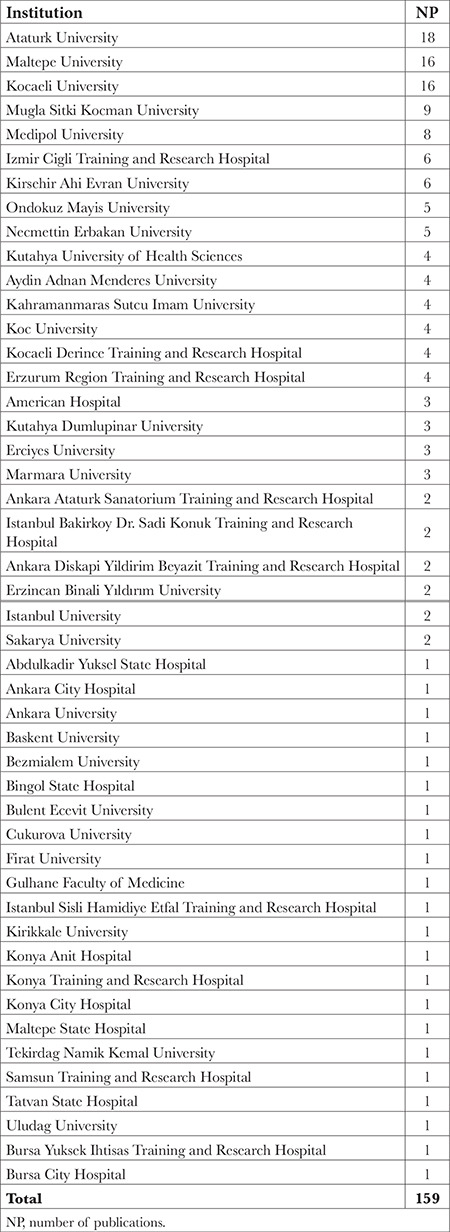
The Institutions Where the First Author Works and the Number of Publications of These Institutions

**Table 4:**
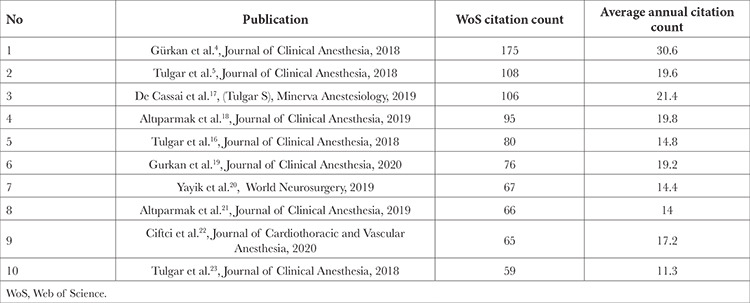
The Top 10 Most Cited Publications, the Number of Citations in the Wos Database and the Annual Average Citation Numbers
